# A Rare Case of Facial Artery Branching—A Review of the Literature and a Case Report with Clinical Implications

**DOI:** 10.3390/medicina57111172

**Published:** 2021-10-28

**Authors:** Martin Siwetz, Nicol Turnowsky, Niels Hammer, Michael Pretterklieber, Andreas Wree, Veronica Antipova

**Affiliations:** 1 Gottfried Schatz Research Center for Cell Signaling, Metabolism and Aging, Macroscopic and Clinical Anatomy, Medical University of Graz, 8010 Graz, Austria; martin.siwetz@medunigraz.at (M.S.); nicol.turnowsky@medunigraz.at (N.T.); niels.hammer@medunigraz.at (N.H.); veronica.antipova@medunigraz.at (V.A.); 2 Department of Trauma, Orthopedic and Plastic Surgery, University Hospital of Leipzig, 04103 Leipzig, Germany; 3 Fraunhofer Institute for Machine Tools and Forming Technology, 09126 Dresden, Germany; 4 Institute of Anatomy, Rostock University Medical Center, 18057 Rostock, Germany; andreas.wree@med.uni-rostock.de

**Keywords:** aesthetic medicine, anatomical variation, branching, face, facial artery, face surgery, flaps, plastic and reconstructive surgery

## Abstract

*Background and Objectives:* Vascular variations appear as morphologically distinct patterns of blood diverging from the most commonly observed vessel patterns. The facial artery is considered to be the main vessel for supplying blood to the anterior part of the face. An anatomical understanding of the facial artery, its course, its topography, and its branches is important in medical and dental practice (especially in neck and face surgery), and is also essential for radiologists to be able to interpret vascular imaging in the face following angiography of the region. A profound knowledge of the arteries in the region will aid in minimizing the risks to the patient. *Materials and Methods:* In our publication a narrative literature review and a case report are presented. *Results:* A rare case of a facial artery pattern has been described anatomically for the first time with respect to its course and branching. This variation was found on the left side of a 60-year-old male corpse during anatomical dissection. The anterior branch of the facial artery arched in the direction of the labial angle, and there divided into the inferior and superior labial arteries. At the same time, the posterior branch coursed vertically and superficially to the masseter muscle. It here gave off the premasseteric branch, and continued towards the nose, where it ran below the levator labii superioris and the levator labii superioris alaeque nasi muscles and terminated at the dorsum nasi. *Conclusions*: Our review of the literature and the case report add to knowledge on the facial artery with respect to its topographical anatomy and its branching and termination patterns, as well as the areas of supply. An exact knowledge of individual facial artery anatomy may play an important role in the planning of flaps or tumor excisions due to the differing vascularization and can also help to prevent artery injuries during aesthetic procedures such as filler and botulinum toxin injections.

## 1. Introduction

The facial artery is a main artery supplying the facial region [1,2,3,4]. It is typically described as a branch of the external carotid artery, where it may form trunks with the lingual and superior thyroid or (in rare cases) with the maxillary artery [5]. It crosses the stylohyoid muscle as well as the posterior belly of the digastric muscle on their medial side [6,7,8,9]. In its further regular course it crosses the mandible anterior to the masseter muscle and ascends in direction to the nose, where it ends in variable forms between the lower lip and the medial angle of the eye [10,11].

The typical branches of the facial artery in the facial region, besides the muscular branches, are the inferior and superior labial branch, the nasal septal branch, the lateral nasal branch, and the angular artery as the terminal branch [10,11,12]. However, a narrative review of the literature revealed considerable variations in the facial artery with respect its branching patterns. In this case report an apparently exceptional division of the facial artery into an anterior and posterior main branch is anatomically described for the first time.

Preoperative knowledge on individual anatomical variations in facial artery patterns may aid maxillofacial surgeons in reconstructive planning to effectively manage facial injuries with arterial involvement, dermatologists in aesthetic procedures, and radiological anatomy professionals in the field of malignancies for the treatment of various facial tumors by embolization [13,14,15,16]. Information on variations in the facial artery may also contribute to damage risk mitigation during cosmetic procedures such as neurotoxin or filler injections [17,18].

## 2. Materials and Methods

### 2.1. Narrative Literature Review

A review of the literature was performed using the PubMed database. Search terms included ‘facial artery’, ‘facial artery AND variations’, ‘facial artery AND branching’ and ‘a. facialis’. Furthermore, books on the human anatomy and the anatomy of the face and arteries as well as handbooks from various clinical disciplines involving the facial region were studied. The results of the narrative literature review are presented in the Results and Discussion section.

### 2.2. Case Report

A rare variation in the course and branching pattern of the facial artery on the left side of a 60-year-old male body donor was observed during the dissection course in anatomy of the Macroscopic and Clinical Anatomy. The specimen was donated to the Division of Macroscopic and Clinical Anatomy of the Medical University of Graz under the approval of the Anatomical Donation Program of the Medical University of Graz and according to the Austrian laws concerning body donations.

The body donor was preserved using an ethanol–glycerin based solution [19]. Upon gross examination, the corpse yielded no facial tumors, signs of previous injury or subsequent surgery, or signs of vascular disease in the facial region. During his lifetime, donor had given written informed consent to participate in anatomical studies.

## 3. Results

### 3.1. Literature Review

A total of 18 studies and reports on the branching pattern of the facial artery were found. An overview of the described patterns and their frequency according to the authors can be found in Table 1.

### 3.2. Case Report

**Figure 1:**
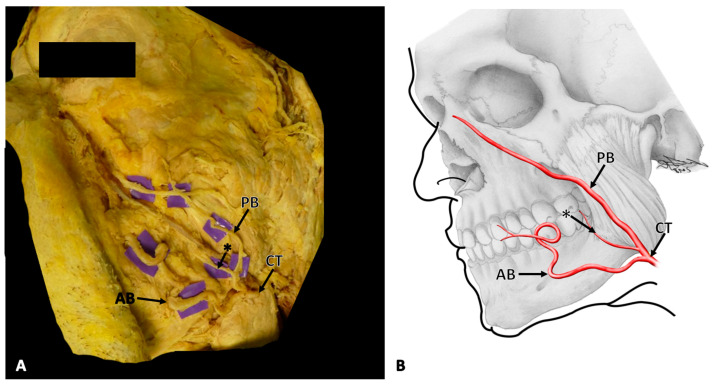
(**A**) The dissection situs of the left side of a male face with a rare variation in the branching pattern of the facial artery. (**B**) A schematic depiction of the dissection situs and branching pattern of the facial artery on the left side of the face in the same orientation. The common trunk (CT) of the facial artery splits at the level of the base of the mandible into an anterior branch (AB) and a posterior branch (PB) that gives off the premasseteric branch (*).

Following skin removal, the facial artery was identified at the anterior aspect of the masseter muscle. Its course was followed proximally and distally, paying special attention not to damage any vascular structures. In this case, at the level of the base of the mandible, the facial artery divided into two distinct branches, namely an anterior and posterior branch (Figure 1A,B).

The anterior branch took a tortuous course towards the labial angle, where it further divided into a superior and an inferior labial artery. The posterior branch coursed more vertically, thereby running superficially to the masseter muscle, where it gave off the premasseteric branch. It continued towards the nose, where it crossed below the levator labii superioris and levator labii superioris alaeque nasi muscles and terminated at the dorsum of the nose. The contralateral facial artery of the specimen showed no variations, with a course and termination that can be best classified as nasal type, similar to types previously described in the literature (see Table 1).

## 4. Discussion

While variations in the branching pattern of the facial artery have been described previously, most of these variations apply to the terminal branching of the artery [11,23], and not to a split of the artery into two main branches. In 1928, Buntaro Adachi described a fine artery branch arising from the facial artery (external maxillary artery) at the level of the mandible following approximately the anterior margin of the masseter muscle. This so-called “premasseteric branch” is described to end or anastomose soon after its origin [29]. The premasseteric branch is also known as the posterior branch of the facial artery and is generally found to terminate in the region of the parotid duct [30].

**Figure 2:**
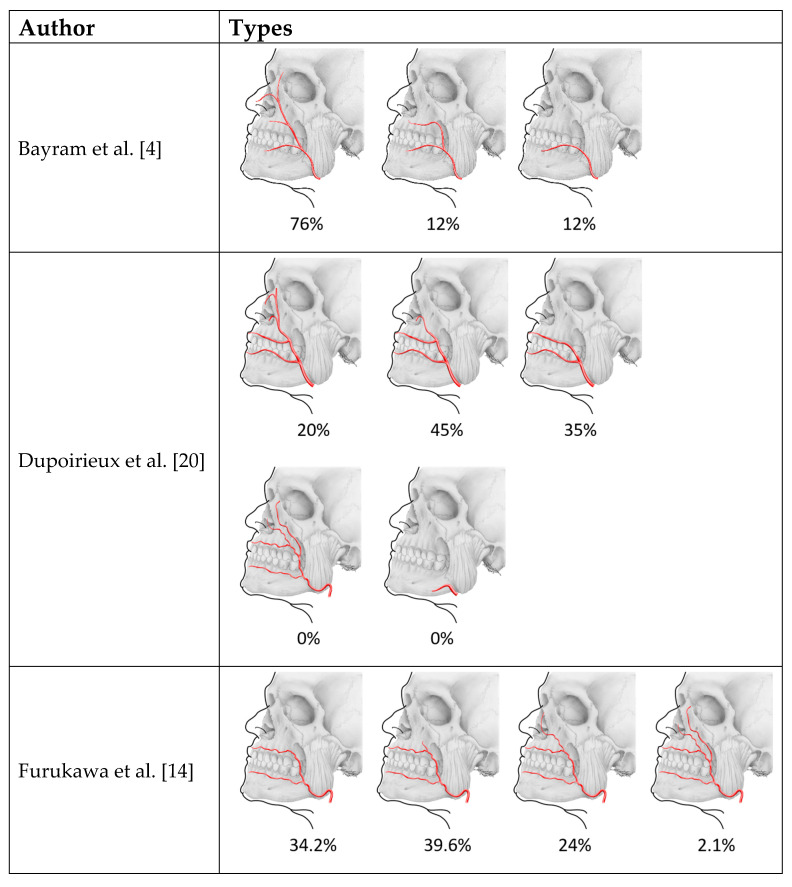

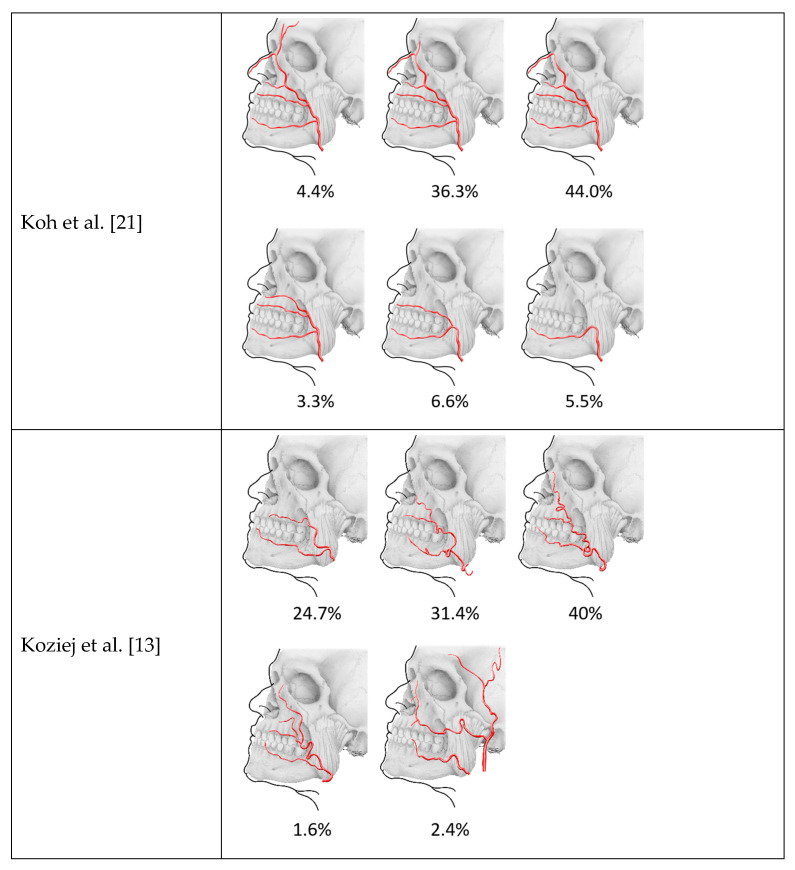

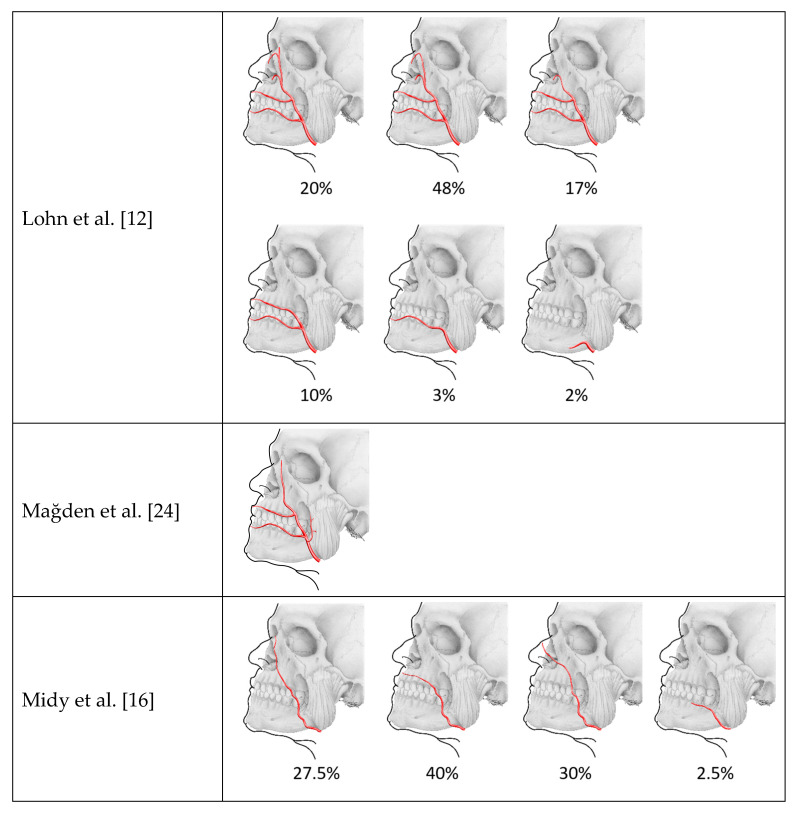

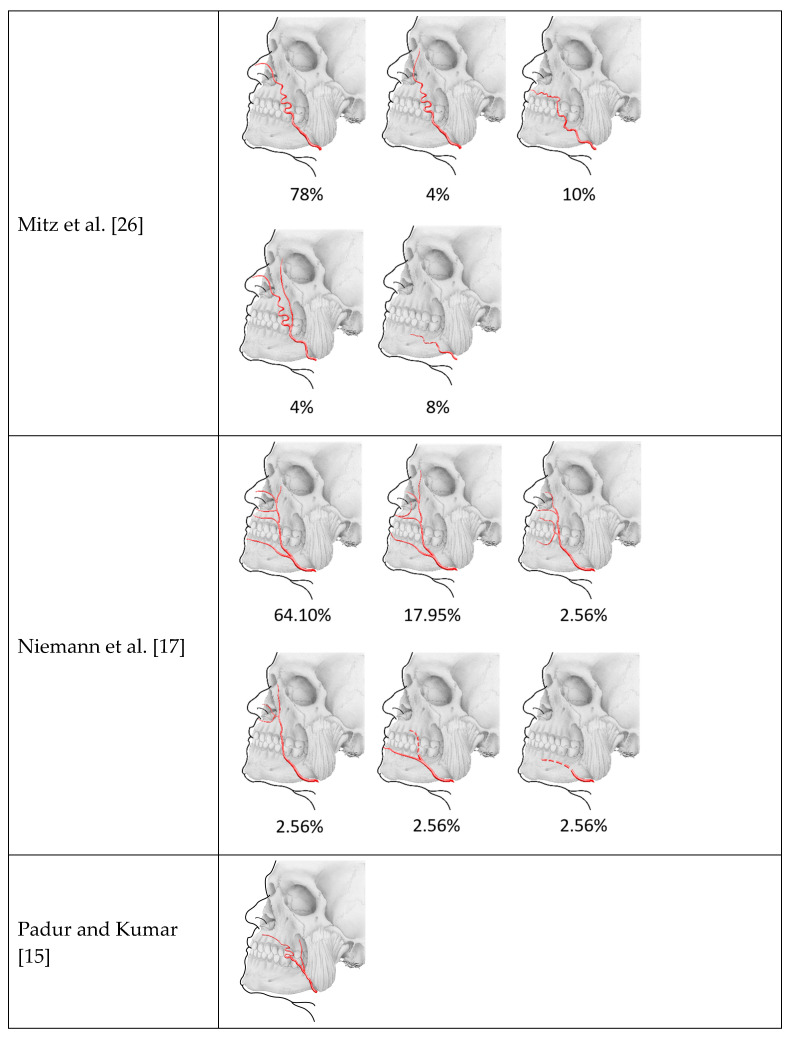

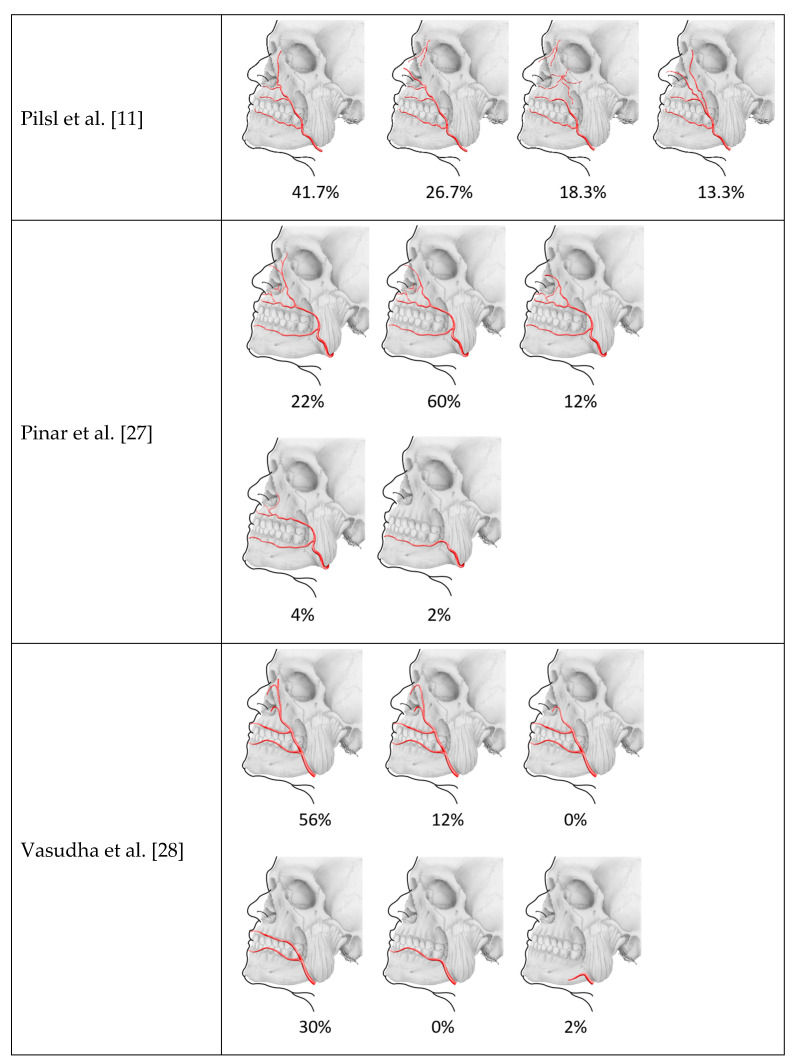
Types of facial artery according to prior literature. Lateral views according to the original depiction.

When evaluating the premasseteric branch of the facial artery, its typical origin was 11.3 mm higher than the basis of the mandible, and its course was found to follow the anterior border of the masseter muscle. The premasseteric branch was shown to have superficial and deep branches, with the superficial branch having a mean length of 27.4 mm and a diameter of 0.9 mm [24] (Table 1, Figure 2).

**Figure 3:**
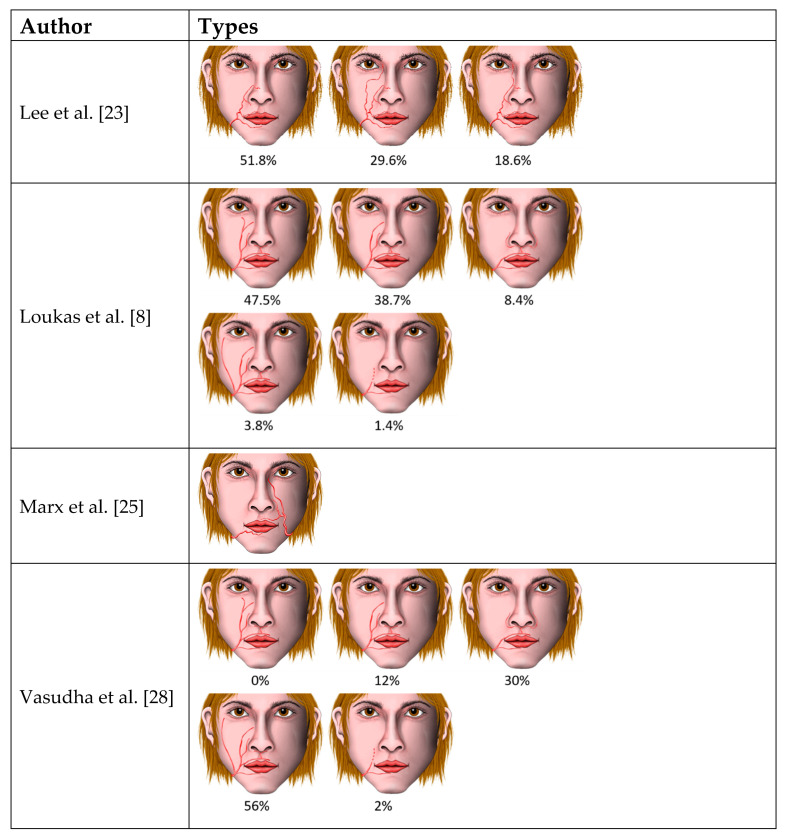
Types of facial artery branching patterns. Frontal views according to the original depiction.

Besides this premasseteric branch, a branching pattern of the facial artery into an anterior and posterior branch was described in 13.3% of cases, whereby the origin of this branch was found after the release of the inferior labial artery, and the posterior branch followed the anterior branch just 10–20 mm behind [11] (Table 1, Figure 2). Furthermore, Loukas et al. [8] described a duplex or a long course of the facial artery, which displayed an angular branch arising individually below the oral commissure directly from the facial arterial trunk, but which terminated as the superior alar artery (Table 1, Figure 3). Their pattern does not correspond to the here-presented findings, since in this case the branching was observed at the level of the base of the mandible and the branches took a completely separated course.

Another variant is described in 2% of cases in which the angular artery originates as a separate branch of the facial artery before the release of the inferior labial artery, but with all other branches (inferior labial, superior labial, inferior alar, and lateral nasal artery) originating from the remaining facial artery after the origin of the angular artery [12] (Table 1, Figure 2). This is also in contrast to our findings, in which the anterior branch ends as the superior labial artery and the branches supplying the nose (inferior alar and lateral nasal artery) are branches of the posterior branch.

Padur and Kumar [15] described a similar case where the facial artery divided into an anterior and posterior branch. The anterior branch terminated as the superior labial artery (as in our case), while in their case the posterior branch terminated in the region of the parotid duct (Table 1, Figure 2). Therefore, this case also does not correspond to the findings presented here since the posterior branch ended at the dorsum of the nose.

Lasjaunias et al. [22]—based on angiographic findings—described the vascularization of the face in a merely qualitative way. A labial trunk supplying the lower and upper lip was part of their findings. This is coherent with our findings regarding the anterior branch of facial artery. Furthermore, they described a jugal trunk as a branch of the facial artery that further divides in a buccomasseteric or buccal artery and a posterior jugal artery. This posterior jugal artery follows the course of the main facial trunk supplying the upper and posterior cheek. Besides differences in methodology, the described jugal artery shows some similarity to the here-presented posterior branch regarding the course of the vessel. However, it differs in termination and area of supply since it was described to supply the upper and posterior cheek, in contrast to the medial cheek and dorsum of the nose in the here-presented case.

Previous anatomical studies that have described the known cases of branching of the facial artery are shown in Table 1 and in Figure 2 and Figure 3.

The knowledge of the anatomy of the facial artery is of special importance for plastic and maxillofacial surgeons in order to reduce the risk of iatrogenic injury to the artery as well as flap necrosis during procedures such as tumor resection (e.g., of squamous cell carcinomas which are often located in the cheek) [31] and lip repair [20,32,33,34]. When creating a commonly used facial artery musculo-mucosal flap the facial artery needs to be identified and dissected. Therefore, knowledge of the branching of the facial artery branching and its area of supply is important to avoid flap necrosis due to avascularisation [20,32,33,34]. Additionally, knowledge of the anatomy of the facial artery is important in aesthetic procedures when fillers are injected, as a risk of blindness or necrosis of parts of the face has been described [35]. Furthermore, radiologists may benefit in the interpretation of facial artery angiography [28]. The present case of the facial artery and the narrative review of the literature (Table 1), as well as the schematic representation of described branching patterns of the facial artery and their frequency from the same publications (Figure 2 and Figure 3), may provide useful information for anatomists and for applications in different fields of clinical practice and surgery.

## 5. Conclusions

The facial artery forms the main vessel that supplies blood to the facial region. The arterial vascular system of the face varies greatly between individuals and even between the left and right halves of the face of the same person.

Therefore, its location and course are important for safe handling in both surgical and non-surgical interventions. To the best of our knowledge, we present here for the first time a case of a very rare type of a facial artery. This information could further support craniofacial surgery and could help improve the quality of treatment. A good knowledge of all variations and anomalous branching patterns is of academic and clinical significance for general practitioners, plastic and maxillofacial surgeons, radiologists, otolaryngologists, and traumatologists.

## Figures and Tables

**Table 1:** Summary of described branching patterns of the facial artery and their frequency (literature review).

Author	Type of Study	Number of Arteries	Types/Categories	Frequency	Annotations
Bayram et al. [4]	Anatomical study	n = 25	3 types based on termination	Type 1 (angular): 76% Type 2 (superior labial): 12% Type 3 (inferior labial): 12%	Study performed on fetuses
Dupoirieux et al. [20]	Anatomical study	n = 20	5 classes based on pattern and termination	Type 1 (angular): 20% Type 2 (nasal dorsal): 45% Type 3 (coronal labial): 35% Type 4 (double): 0% Type 5 (weak): 0%	
Furukawa et al. [14]	Head and neck CT with contrast agent	n = 187	4 types based on termination	Type 1 (superior labial): 34.2% Type 2 (nasolabial): 39.6% Type 3 (lateral nasal): 24% Type 4 (dominant angular): 2.1%	
Koh et al. [21]	Anatomical study	n = 91	6 types based on the pattern of final arterial branches	Forehead type: 4.4% Angular type: 36.3% Nasal type: 44% Alar type: 3.3% Superior labial type: 6.6% Inferior labial type: 5.5%	
Koziej et al. [13]	Head and neck CT with contrast agent	n = 255	5 categories based on termination and branching	Type I (proximal to superior labial): 24.7% Type II (distal to superior labial): 31.4% Type III (textbook course): 40% Type IV (dominant lateral angular): 1.6% Type V (hypoplastic vessel): 2.4%	
Lasjaunias et al. [22]	Angiographic findings		Qualitative description of the vascularization of the face and branches of the facial artery and their hemodynamic important anastomoses	Functional arterial monopedicles formed by the following (partly) anastomosing branches: Ascending palatine artery Submaxillary artery Submental artery Inferior masseteric artery Jugal trunk (buccal artery and posterior jugal artery) Middle mental artery Inferior labial artery Superior labial artery Anterior jugal artery Nasal arcade Angular orbital artery	
Lee et al. [23]	Anatomical study	n = 54	3 categories based on the branching	Type I (nasolabial type): 51.8% Type II (infraorbital trunk): 29.6% Type III (forehead pattern): 18.6%	
Lohn et al. [12]	Anatomical study	n = 201	6 categories based on the terminal branch	Type I (angular): 20% Type II (lateral nasal): 48% Type III (inferior alar): 17% Type IV (superior labial): 10% Type V (inferior labial): 3% Type VI (undetected): 2%	
Loukas et al. [8]	Anatomical study	n = 200	6 types based on the branching and termination with subtypes	Type A (typical arrangement): 47.5% Type B (nasal): 38.7% Type C (superior labial–alar): 8.4% Type D (duplex or long course): 3.8% Type E (rudimentary): 1.4%	Duplex, being an angular artery branching off at the level of the mouth
Mağden et al. [24]	Anatomical study	n = 27	Neurovascular and anatomical features and relations of the premasseteric branch and its branches (origin location, diameter, length, course)	Separate origin of the premasseteric branch from the facial artery in all cases Location determined according to the body of the mandible Diameter 1.12 mm (at level of origin), in 3% of cases larger than the facial artery Observation of the course up to the upper anterior border of the masseter Description of branches and anastomoses	
Marx et al. [25]	Case report				Anomalous artery pattern in both sides Termination as inferior labial on the right, missing inferior labial on the left
Midy et al. [16]	Anatomical study	n = 40	4 types based on the termination	Type 1 (angular): 27.5% Type 2 (labial): 40% Type 3 (nasal): 30% Type 4 (abortive): very uncommon, found only once	
Mitz et al. [26]	Anatomical study	n = 50	5 types based on course, pattern, and termination	Type 1 (nasal): 78% Type 2 (classic): 4% Type 3 (intermediate): 10% Type 4 (duplicate): 4% Type 5 (weak): 8%	
Niemann et al. [17]	Anatomical study	n = 39	6 types based on the origin of the branches	Type 1, each of the branches arose separately from the facial artery, as in the standard anatomic definition: 64.10% Type 2, the superior labial branch gave off an inferior alar branch: 17.95% Type 3, the superior and inferior labial branches originated from a common trunk and the facial artery had early termination: 2.56% Type 4, the superior and inferior alar branches came from a common trunk and no superior labial and inferior labial branches were present: 2.56% Type 5, the facial artery was rudimentary, which is when the artery terminated after giving off an inferior labial artery, but before reaching the upper lip: 2.56% Type 6, the facial artery was abortive, which is when the facial artery gives off no facial branches: 2.56%	
Padur and Kumar [15]	Case report				Bifurcation of the facial artery with posterior branch terminating in the buccal region
Pilsl et al. [11]	Anatomical study	n = 60	4 types based on course, pattern, and termination	Type 1 (textbook course): 41.7% Type 2 (no angular): 26.7% Type 3 (superior labial): 18.3% Type 4 (two branches): 13.3%	In type 4, the branching was observed at the level of the mouth after release of the inferior labial artery
Pinar et al. [27]	Anatomical study	n = 50	5 classes based on termination	Type 1 (angular): 22% Type 2 (nasal): 60% Type 3 (alar): 12% Type 4 (superior labial): 4% Type 5 (hypoplastic): 2%	
Vasudha et al. [28]	Anatomical study	n = 50	Classification based on description of Koh et al., Bayram et al. and Loukas et al.	Type 1 (angular): 56% Type 2 (superior labial): 30% Type 3 (inferior labial): 0% Type A (typical arrangement): 0% Type B (nasal): 12% Type C (superior labial—alar): 30% Type D (duplex or long course): 56% Type E (rudimentary): 2% Type I (angular): 56% Type II (lateral nasal): 12% Type III (inferior alar): 0% Type IV (superior labial): 30% Type V (inferior labial): 0% Type VI (undetected): 2%	

## Data Availability

Not applicable.
